# EGFRvIII upregulates DNA mismatch repair resulting in increased temozolomide sensitivity of *MGMT* promoter methylated glioblastoma

**DOI:** 10.1038/s41388-020-1208-5

**Published:** 2020-02-17

**Authors:** Nina Struve, Zev A. Binder, Lucy F. Stead, Tim Brend, Stephen J. Bagley, Claire Faulkner, Leonie Ott, Justus Müller-Goebel, Anna-Sophie Weik, Konstantin Hoffer, Leonie Krug, Thorsten Rieckmann, Lara Bußmann, Marvin Henze, Jennifer J. D. Morrissette, Kathreena M. Kurian, Ulrich Schüller, Cordula Petersen, Kai Rothkamm, Donald M. O´ Rourke, Susan C. Short, Malte Kriegs

**Affiliations:** 1 Laboratory of Radiobiology & Experimental Radiation Oncology, Hubertus Wald Tumorzentrum – University Cancer Center Hamburg, University Medical Center Hamburg-Eppendorf, Hamburg, Germany; 2 Department of Neurosurgery, Perelman School of Medicine at the University of Pennsylvania, Philadelphia, PA USA; 3 Leeds Institute of Medical Research at St James’s, Wellcome Trust Brenner Building, St. James’s University Hospital, Leeds, UK; 4 Division of Hematology/Oncology, Department of Medicine, Perelman School of Medicine at the University of Pennsylvania, Philadelphia, PA USA; 5 Bristol Genetics Laboratory, Southmead Hospital, Bristol, UK; 6 Department of Otorhinolaryngology and Head and Neck Surgery, University Medical Center Hamburg Eppendorf, Hamburg, Germany; 7 Department of Radiotherapy and Radiooncology, University Medical Center Hamburg-Eppendorf, Hamburg, Germany; 8 Division of Precision and Computational Diagnostics, Department of Pathology and Laboratory Medicine, Hospital of the University of Pennsylvania, Philadelphia, PA USA; 9 Bristol Brain Tumour Research Centre, University of Bristol, Bristol, UK; 10 Research Institute Children’s Cancer Center Hamburg, Hamburg, Germany; 11 Institute of Neuropathology, University Medical Center Hamburg-Eppendorf, Hamburg, Germany; 12 Department of Pediatric Hematology and Oncology, University Medical Center Hamburg-Eppendorf, Hamburg, Germany

**Keywords:** DNA mismatch repair, CNS cancer, Cell signalling

## Abstract

The oncogene epidermal growth factor receptor variant III (EGFRvIII) is frequently expressed in glioblastomas (GBM) but its impact on therapy response is still under controversial debate. Here we wanted to test if EGFRvIII influences the sensitivity towards the alkylating agent temozolomide (TMZ). Therefore, we retrospectively analyzed the survival of 336 GBM patients, demonstrating that under standard treatment, which includes TMZ, EGFRvIII expression is associated with prolonged survival, but only in patients with *O6-methylguanine-DNA methyltransferase* (*MGMT*) promoter methylated tumors. Using isogenic GBM cell lines with endogenous EGFRvIII expression we could demonstrate that EGFRvIII increases TMZ sensitivity and results in enhanced numbers of DNA double-strand breaks and a pronounced S/G2-phase arrest after TMZ treatment. We observed a higher expression of DNA mismatch repair (MMR) proteins in EGFRvIII+ cells and patient tumor samples, which was most pronounced for MSH2 and MSH6. EGFRvIII-specific knockdown reduced MMR protein expression thereby increasing TMZ resistance. Subsequent functional kinome profiling revealed an increased activation of p38- and ERK1/2-dependent signaling in EGFRvIII expressing cells, which regulates MMR protein expression downstream of EGFRvIII. In summary, our results demonstrate that the oncoprotein EGFRvIII sensitizes a fraction of GBM to current standard of care treatment through the upregulation of DNA MMR.

## Introduction

Glioblastoma (GBM, glioma WHO grade IV) is the most common malignant brain tumor in humans. Despite intense treatment including surgery, radio- and chemotherapy the median overall survival time of patients with GBM is less than two years, with huge variations between different subtypes [1, 2]. GBM are characterized by diverse genetic alterations, such as mutations in the *isocitrate dehydrogenase (IDH)* 1 or 2 genes, methylation of the *O6-methylguanine-DNA methyltransferase (MGMT)* promoter, the amplification of the gene encoding the epidermal growth factor receptor (EGFR) or the expression of the EGFR variant III (EGFRvIII). While it is well accepted that *IDH* status and *MGMT* promoter status have prognostic significance in predicting patient survival after standard therapy [3–5], the influence of *EGFR* amplification and EGFRvIII expression on patient survival is still under controversial debate. To address this issue we performed a retrospective survival analysis of GBM patients with known IDH, MGMT and EGFR/EGFRvIII status, who had been treated with surgery, irradiation and TMZ.

The EGFRvIII is caused by an in-frame deletion of *EGFR* exons 2–7. It is predominantly associated with an *EGFR* amplification and is expressed in approximately one-third of all primary GBM [6, 7]. It is localized in clusters within the cellular membrane [8] and shows constitutive tyrosine kinase activity causing an upregulation of pro-survival downstream signaling pathways, such as PI3K/AKT and STAT3 signaling [9, 10]. The EGFRvIII is accepted to be an oncogene and its expression is therefore assumed to have a negative impact on treatment outcome of GBM patients [11–13]. However, clinical studies have so far failed to prove that EGFRvIII is a reliable prognostic marker: while in smaller studies EGFRvIII expression was found to be associated with either better or worse survival, larger studies did not observe any predictive or prognostic impact of EGFRvIII expression [12, 14–19]. A summary of the relevant literature is given in supplementary Table S1 (Table S1). In a previous study using isogenic GBM cells with differences in endogenous EGFRvIII expression we had demonstrated that EGFRvIII has no impact on cellular radiosensitivity [20]. These results are supported by clinical studies of GBM patients treated with adjuvant radiotherapy only [21, 22]. However, today’s standard therapy also includes concurrent TMZ treatment. Because there is no correlation of radiosensitivity and TMZ sensitivity [23], these previous studies do not indicate whether EGFRvIII influences TMZ sensitivity or not. Yet, due to the recent and ongoing development and clinical assessment of various EGFR and EGFRvIII-targeting strategies, such as peptide-based vaccines (rindopepimut), monoclonal antibody immunotoxin conjugates (ABT-414) or EGFRvIII-specific chimeric antigen receptor (CAR) T cells a deeper understanding of the molecular biology and clinical relevance of EGFR amplification and EGFRvIII in GBM is definitely required [24–27].

Here we analyzed if EGFRvIII has an impact on TMZ sensitivity in GBM patients treated with radiotherapy and TMZ. We show here that EGFRvIII expression is specifically associated with prolonged survival, but only if the tumors were *MGMT* promoter methylated. In line with the clinical observation, we observe increased TMZ sensitivity of EGFRvIII expressing and *MGMT* promoter methylated GBM cells in vitro and in vivo*.* The increased TMZ sensitivity could be linked to an increased MMR protein expression and therefore upregulated MMR in EGFRvIII+ cells and tumors.

Regarding cellular signaling, our data show that the increased expression of MMR proteins is mediated by p38 and ERK1/2 downstream of EGFRvIII. These data demonstrate that the oncoprotein EGFRvIII does not mediate resistance but confers sensitivity towards current GBM chemotherapy with TMZ. We, therefore, identified EGFRvIII as a prognostic biomarker, which may point towards an Achilles´ heel in a subset of GBM, warranting further preclinical and clinical studies.

## Results

### EGFRvIII expression is associated with better response to standard of care treatment

**Fig. 1:**
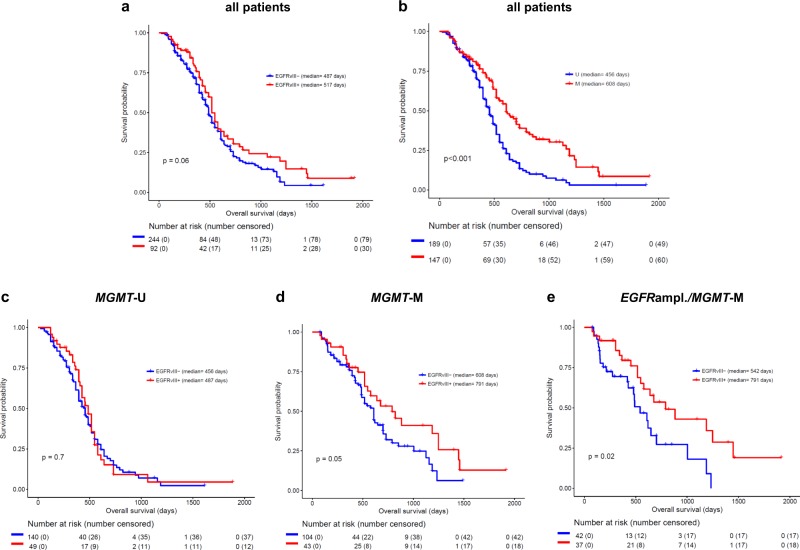
Overall survival according to EGFRvIII, *MGMT* promoter methylation and *EGFR*_ampl._ status. Kaplan–Meier estimates of EGFRvIII−/+ GBM patients treated with standard of care. **a** Entire cohort stratified for EGFRvIII status. **b** Entire cohort stratified for *MGMT* promoter methylation status. **c** *MGMT*-U patients stratified for EGFRvIII status. **d** *MGMT*-M patients stratified for EGFRvIII status. **e** *EGFR*_ampl._/*MGMT*-M patients stratified for EGFRvIII status.

To test if EGFRvIII has an influence on TMZ sensitivity we retrospectively analyzed the overall survival of 336 patients with *IDH1* wild type (wt) GBM and known *MGMT* promoter, *EGFR* amplification and EGFRvIIII status who had been treated with standard of care. Patients from three different cohorts were included, namely the UPenn cohort [28], the Bristol cohort [18] and the TCGA cohort [29] (Table S2). We initially fitted a univariate cox proportional hazards model to include the variables of EGFRvIII status, *EGFR* amplification status, *MGMT* promoter methylation status, gender and age. As expected, we observed a significant association with survival for *MGMT* promoter methylation status (Fig. 1a; hazard ratio [HR] = 0.52; 95% CI = 0.39–0.68; *p* < 0.0001), and age (Fig. S1; HR = 1.02; 95% CI = 1.01–1.04; *p* = 0.002), demonstrating shorter survival for elderly patients. We also detected an association with EGFRvIII status that was significant, albeit at the 10% level (Fig. 1b; HR = 0.75; 95% CI = 0.56–1.02; *p* = 0.06). Using multivariate models including these three variables we observed that age and MGMT promoter methylation status increased in significance with the same HRs, 95% CIs and *p*-values < 0.05. However, the strength of association for EGFRvIII decreased (HR = 0.78; 95% CI = 0.58–1.06; *p* = 0.11). Additional pairwise models indicated that this was owing to an interaction between EGFRvIII and MGMT promoter methylation status, so we proceeded to examine the effect of EGFRvIII in patients stratified as *MGMT* promoter unmethylated (MGMT-U) and *MGMT* promoter methylated (MGMT-M) separately. In *MGMT*-U patients, the EGFRvIII status had no impact on patient survival (Fig. 1c; Fig. S2). For patients with *MGMT*-M tumors, however, EGFRvIII positivity was associated with a median survival benefit of 6 month (791 days vs. 608 days) when compared to the survival of patients with EGFRvIII negative (EGFRvIII-) tumors (Fig. 1d, Fig. S2). Since EGFRvIII expression is associated with *EGFR* amplification [16, 30] (Table S2), we also analyzed the effect of EGFRvIII-positivity solely in patients with *MGMT*-M and *EGFR*ampl tumors. Here, EGFRvIII-positivity was also associated with a favorable outcome (542 days vs. 791 days), with the difference reaching statistical significance (Fig. 1e, Fig. S3; *p* = 0.02) (Fig. 1e; Fig. S3). The beneficial impact of EGFRvIII even remained when analyzing EGFRampl patients irrespective of their MGMT status, which further underpins the findings, that EGFRvIII and not *EGFR* amplification is associated with prolonged survival (Fig. S4). According to this retrospective clinical data set, which includes MGMT-, EGFR- and EGFRvIII-status, EGFRvIII positivity but not *EGFR* amplification is a positive predictor of response to standard of care first line treatment in *MGMT*-M GBM patients.

### EGFRvIII increases cellular TMZ sensitivity

**Fig. 2:**
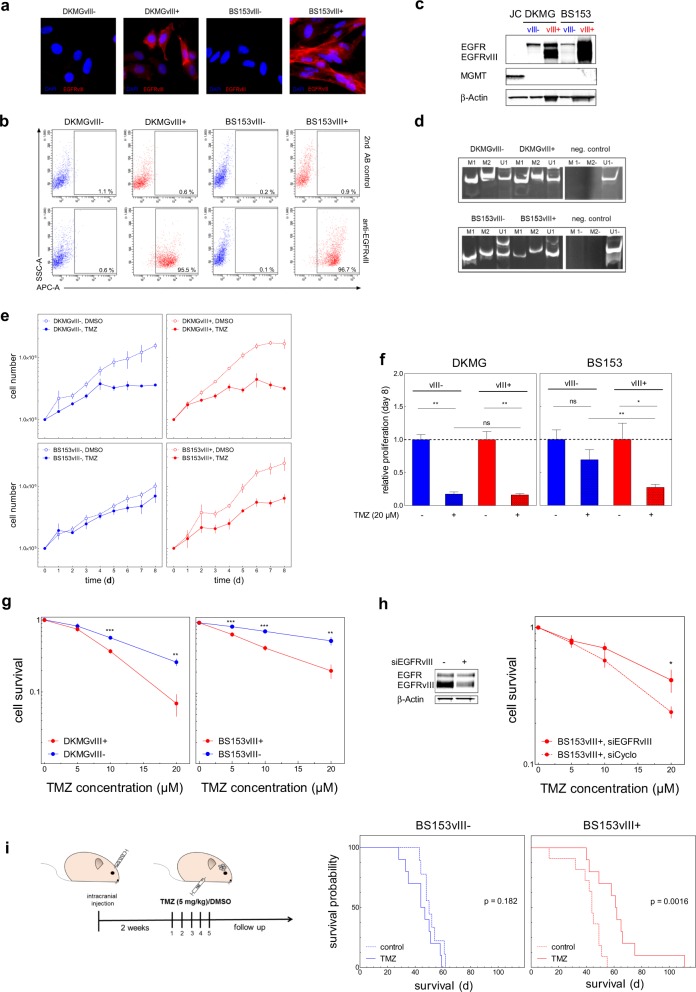
TMZ sensitivity of EGFRvIII−/+ GBM cells and experimental tumors. DKMGvIII−/+ and BS153vIII−/+ human GBM cell lines were used for further analysis. **a** EGFRvIII-specific immunofluorescence staining (red). Cell nuclei were counterstained with DAPI. **b** EGFRvIII expression was detected by flow cytometry using an EGFRvIII-specific antibody (L8A4). A secondary antibody control was used to assess unspecific staining. **c** Detection of EGFR, EGFRvIII, and MGMT by western blot analysis. MGMT expressing Jurkat cells (JC) served as a positive and β-actin as loading control. **d** Analysis of MGMT promoter methylation by PCR. M1, and M2 delineate reactions with methylation-specific primers, U1 with primers for unmethylated DNA. **e** Analysis of proliferation. DKMGvIII−/+ and BS153vIII−/+ cells were treated 24 h after seeding with 20 µM TMZ. Cell number was determined up to 8 days (mean with S.E.M, *n* = 5). **f** Cell growth after 7 days of TMZ treatment. DMSO served as control. The mean value of treated cells was normalized to the mean value of untreated cells (mean with S.E.M, *n* = 5; *P*-values are obtained by two-tailed Student’s *t* test, *p < 0.05, **p < 0.01). **g** Survival of DKMGvIII−/+ and BS153vIII−/+ cells after TMZ treatment assessed by colony forming assay (mean with S.E.M, *n* = 4; *P*-values are obtai*n*ed by two-tailed Student’s *t* test, *p < 0.05, **p < 0.01, ***p < 0.001). **h** Relative cell survival of BS153vIII+ after siRNA-mediated EGFRvIII knockdown. An siRNA against cyclophilin B served as a control (mean with S.E.M, *n* = 4; one-tailed Student’s *t* test was, *p < 0.05, **p < 0.01, ***p < 0.001). **i** Xenograft tumor response to TMZ. Two weeks after the intracranial injection of 2.5 × 10^5^ BS153vIII−/+ cells the mice were treated with 5 mg/kg/d TMZ or solvent for five days. Graph: Kaplan–Meier estimates of survival.

The clinical data indicate an increased sensitivity of EGFRvIII+ GBM cells towards TMZ. We, therefore, tested the impact of EGFRvIII expression on cellular treatment response by using two pairs of isogenic cell lines with (EGFRvIII+) and without (EGFRvIII−) endogenous EGFRvIII expression (Fig. 2a–c) which were established from parental DKMG and BS153 cell lines as described previously [20]. Importantly, neither cell line expressed MGMT (Fig. 2c) due to *MGMT* promoter methylation (Fig. 2d) and MGMT expression was not induced by TMZ treatment (Fig. S5).

Analyzing the proliferation of the EGFRvIII+ and EGFRvIII− sub-cell lines under treatment with clinically relevant TMZ concentrations [31], we observed inhibited cell growth in all cultures (Fig. 2e). There was no difference detectable for the EGFRvIII+ and EGFRvIII- sub-cell lines derived from DKMG (Fig. 2e, f), but for those originating from BS153, EGFRvIII+ cells showed stronger inhibition (Fig. 2e, f). Colony formation assays offer a more stringent analysis of cellular cytotoxicity and here, we indeed observed significant differences for both, the DKMG and BS153 sub-cell lines with EGFRvIII+ cells demonstrating a clear and significant reduction in survival after TMZ treatment (Fig. 2g). Partial knockdown of EGFRvIII in BS153vIII+ cells restored TMZ resistance, confirming that EGFRvIII expression is responsible for increased TMZ sensitivity (Fig. 2h). Furthermore, we tested whether EGFRvIII expression caused increased TMZ sensitivity in vivo, using orthotopic BS153vIII−/+ xenograft tumors grown from BS153 cell cultures with over 90% EGFRvIII positivity and negativity, respectively (Fig. S6). While we observed no significant difference in survival between the untreated BS153vIII− and BS153vIII+ control groups (Fig. S7), TMZ treatment significantly prolonged survival for EGFRvIII+ (44 d vs. 61.5 d) but not EGFRvIII− mice (50 d vs. 45.5; Fig. 4i). Together, these data clearly show that EGFRvIII expression is associated with cellular TMZ sensitivity, which is further demonstrated by improved tumor response in vivo.

### EGFRvIII increases DNA damage after TMZ treatment due to upregulated MMR

**Fig. 3:**
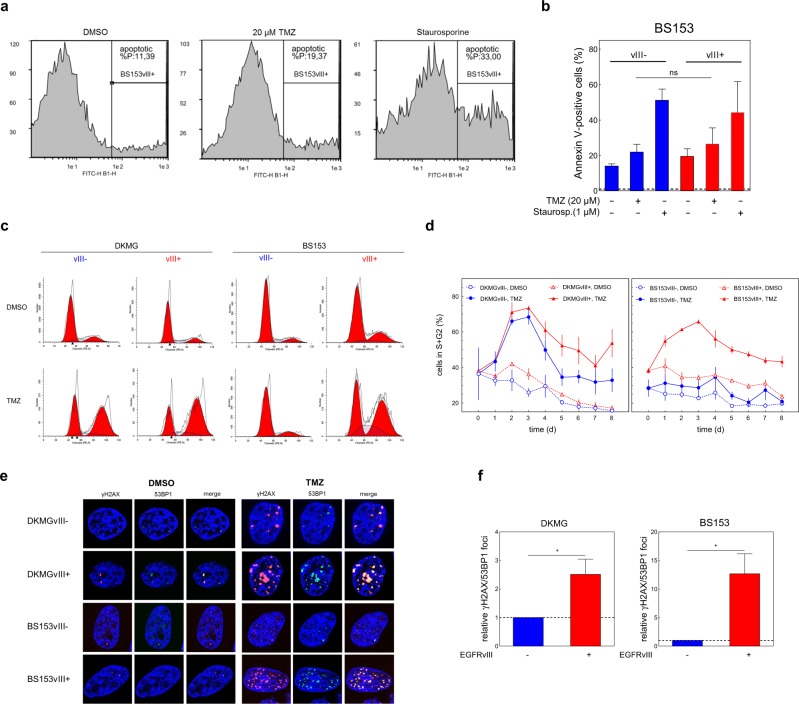
Apoptosis, cell cycle distribution, and DNA repair after TMZ treatment. **a** Representative flow cytometry analysis data from Annexin V-FITC staining of BS153vIII+ cells after TMZ treatment. Cells were treated with 20 µM TMZ for 96 h or 1 µM staurosporine as a positive control for 12 h. DMSO was used as a control. **b** Quantification of Annexin V-positive BS153vIII−/+ cells after TMZ and staurosporine treatment (mean with S.E.M, *n* = 3; one-tailed Student’s *t* test: ns = not significant). **c** Representative propidium iodide (PI)-stained DNA content profiles 3 d after TMZ and DMSO treatment. **d** G2/S content in DKMGvIII−/+ and BS153vIII−/+ cells after TMZ and DMSO treatment analyzed by PI staining and flow cytometry (mean with S.E.M, *n* = 5). **e** Detection of DNA DSB in DKMGvIII−/+ and BS153vIII−/+ cells 3 d after TMZ treatment using immunofluorescence staining of γH2AX/53BP1 co-localized foci. **f** Quantification of relative γH2AX/53BP1 co-localized foci in TMZ-treated DKMGvIII−/+ and BS153vIII−/+ cells. Cell nuclei were counterstained with DAPI (mean with S.E.M, DKMGvIII−/+: *n* = 4; BS153vIII−/+: *n* = 5; *P*-values are obtained by one-tailed Student’s *t* test, *p < 0.05).

Analysis of Annexin V positive cells as a marker for apoptosis revealed no difference between EGFRvIII− and EGFRvIII+ BS153 cells although 20 µM TMZ induced apoptosis in a fraction of cells (Fig. 3a, b). This demonstrates that increased inactivation of EGFRvIII+ cells after 20 µM TMZ treatment is caused by apoptosis-independent mechanisms.

When analyzing the cell cycle distribution we detected an increase in the fraction of S/G2-phase cells for the EGFRvIII+ sub-cell lines (Fig. 3c, d, Fig. S8), which was more pronounced for the BS153 cells. Since such an S/G2-arrest often indicates accumulation of DNA damage and increased replication stress we analyzed the DNA double-strand break (DSB) marker proteins γH2AX and 53BP1 after TMZ treatment. In both EGFRvIII+ cell lines we observed an increase in the number of γH2AX/53BP1 double-positive foci after TMZ treatment compared to their EGFRvIII− counterparts, demonstrating more TMZ-induced DSB (Fig. 3e, f).

**Fig. 4:**
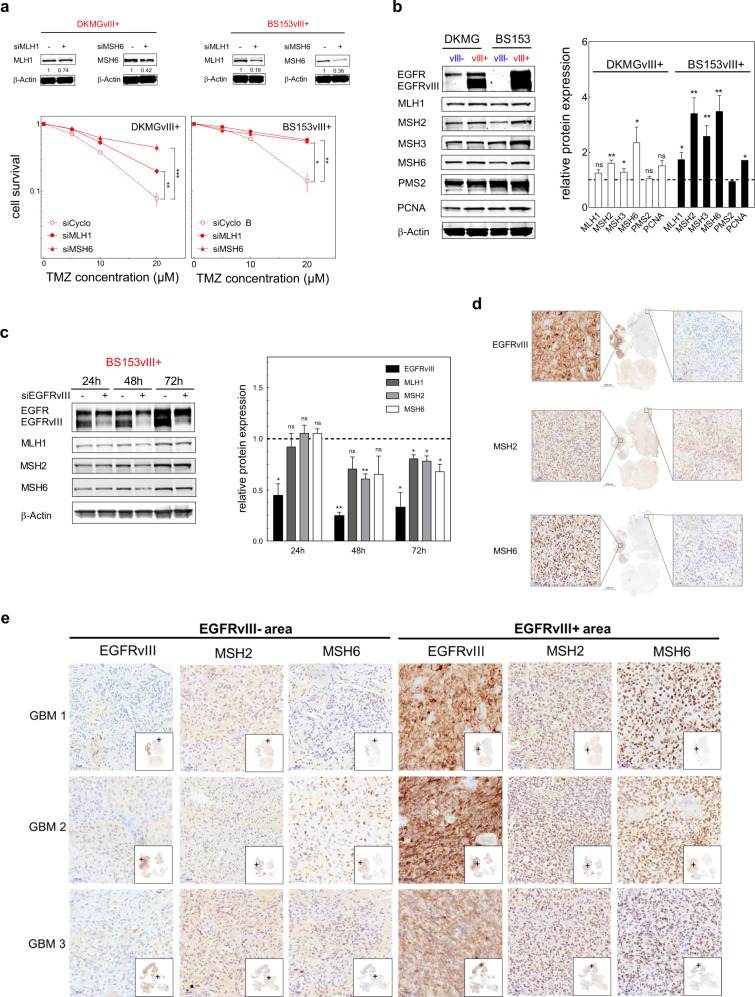
MMR protein expression in vitro and in situ. **a** Impact of siRNA-mediated reduction of MMR protein expression in DKMGvIII+ and BS153vIII+ cells. Upper Western blots: Confirmation of MLH1 and MSH6 knockdown. Bottom: Clonogenic survival after TMZ treatment. An siRNA against cyclophilin B served as a control. (mean with S.E.M, *n* = 3; *P* values are obtained by one-tailed Student’s *t* test, *p < 0.05, **p < 0.01, ***p < 0.001). **b** Expression of MMR proteins and PCNA in DKMGvIII−/+ and BS153vIII−/+ cells. For western blot analysis, samples were normalized to cell number. β-actin served as loading control. For quantification of MMR protein and PCNA expression the relative expression values of EGFRvIII+ cells were normalized to the relative values of EGFRvIII- cells (mean with S.E.M, *n* = 3; *P*-values are obtained by one-tailed Student’s *t* test, *p < 0.05, **p < 0.01). **c** Impact of siRNA-mediated EGFRvIII knockdown in BS153vIII+ cells on MLH1, MSH2, and MSH6 expression after 24, 48, and 72 h. An siRNA against cyclophilin B served as a control. Western blot analysis, β-actin served as a control. For quantification of relative MMR protein expression protein levels were normalized to β-actin and to MLH1, MSH2, and MSH6 levels in the control (mean with S.E.M, *n* = 5; *P*-values are obtained by one-tailed Student’s *t* test, *p < 0.05, **p < 0.01). **d** Immunohistochemical detection of EGFRvIII, MSH2 und MSH6 expression in one representative GBM patient sample (GBM1) displaying heterogeneous EGFRvIII expression. Scale bars represent 50 and 5000 µm (overview in the middle). **e** Immunohistochemical detection of EGFRvIII, MSH2 und MSH6 expression in three GBM patient samples displaying heterogeneous EGFRvIII expression. EGFRvIII− negative areas are depicted on the left, EGFRvIII+ on the right. Scale bars represent 50 µm.

TMZ induces DSB by a futile MMR cycle: if TMZ-induced O6meG cannot be repaired by MGMT, MMR removes the incorrectly paired thymidine, leading to the accumulation of DSB during replication [32, 33]. Therefore, enhanced MMR activity leads to an increase in TMZ-induced DSB, resulting in increased cellular sensitivity towards TMZ and other alkylating agents [34–39]. Since MMR activity, and thereby TMZ sensitivity, can be regulated by the level of MMR protein expression [37], we next analyzed MMR protein expression in EGFRvIII+ and EGFRvIII− cells in detail. First, we confirmed the importance of MMR protein expression for TMZ sensitivity in DKMGvIII+ and BS153vIII+ cells by partially knocking down the key MMR proteins MLH1 and MSH6 (Fig. 4a). As expected, cells became TMZ resistant if the expression of MLH1 or MSH6 was reduced. Even a moderate 20–60% reduction in protein expression was sufficient to confer a pronounced TMZ resistance, which is in line with the observation of McFaline-Figueroa et al. [37]. Overall, the downregulation of MLH1 and MSH6 was more efficient in BS153vIII+ compared to DKMGvIII+ cells, leading to almost complete TMZ resistance in these cells. These data demonstrate that endogenous EGFRvIII expression specifically sensitizes MGMT deficient cells to TMZ treatment by upregulating MMR.

When analyzing the expression of MMR proteins in EGFRvIII− and EGFRvIII+ cells we observed an elevated expression of MMR proteins in both EGFRvIII+ cell lines, which was statistically significant for MSH2, MSH3, and MSH6 in DKMGvIII+ and for all detected MMR proteins in BS153vIII+ (Fig. 4b). Vice versa, siRNA-mediated downregulation of EGFRvIII in BS153vIII+ cells resulted in a significant decrease in MMR protein expression as measured 72 h after siRNA treatment (Fig. 4c).

To further validate these findings we assessed MSH2 and MSH6 expression in situ using samples from three individual GBM patients, each displaying heterogonous EGFRvIII expression. The samples were randomly chosen from an independent cohort. We observed indeed a pronounced difference between EGFRvIII− and EGFRvIII+ areas. MSH2 and MSH6 expression were clearly upregulated in EGFRvIII+ areas, thereby corroborating our in vitro findings (Fig. 4d, e).

Taken together, these data strongly suggest that endogenous EGFRvIII expression specifically sensitizes MGMT-deficient cells to TMZ treatment by upregulating MMR.

### EGFRvIII regulates MMR protein expression via MAPK-signaling

**Fig. 5:**
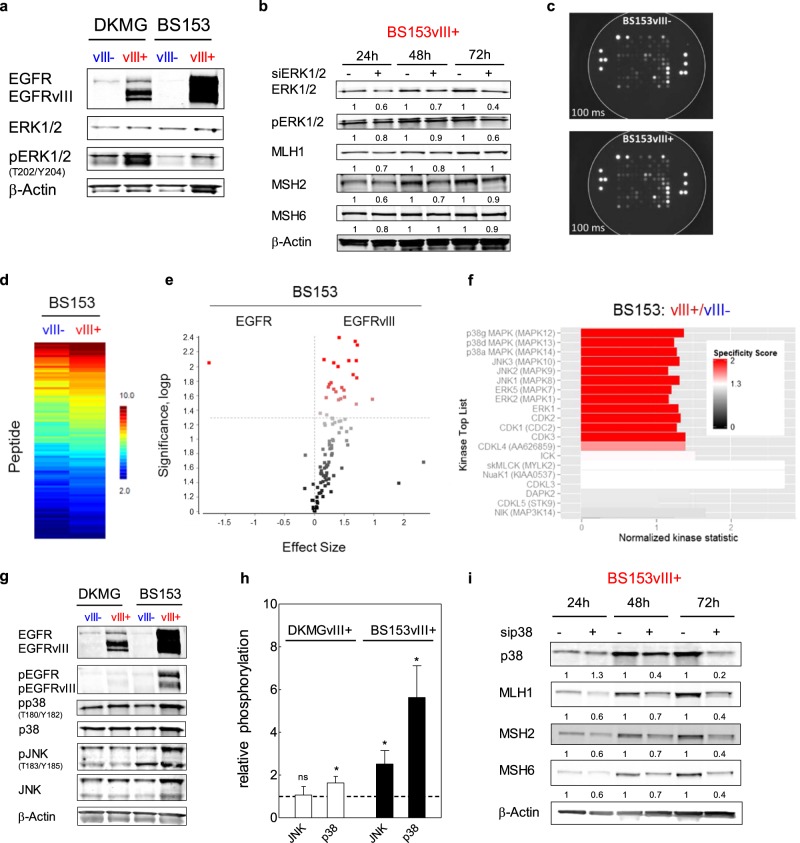
Impact of MAPK signaling on MMR protein expression. **a** Detection of EGFR, EGFRvIII, ERK1/2, pERK1/2 (T202/Y204) by western blot analysis. β-actin served as loading control. Samples were normalized to cell number. **b** Impact of siRNA mediated ERK1/2 knockdown in BS153vIII+ cells on MLH1, MSH2, and MSH6 expression after 24, 48, and 72 h. An siRNA against cyclophilin B served as a control. Western blot analysis, β-actin served as a control. For quantification of relative MMR protein expression protein levels were normalized to β-actin and to MLH1, MSH2, and MSH6 levels in the control. **c–f** Functional kinome profiling. **c** Detection of sequence-specific peptide phosphorylation on STK-PamChip® using anti-phospho-Ser/Thr antibodies (cycle: 124; exposure time: 100 ms). **d** Heatmap showing log2-transformed signal intensities for the phosphorylated peptides. The signals were sorted from high (red) to low (blue) intensity/phosphorylation. **e** Two-group comparison of EGFRvIII+ vs. EGFRvIII− depicted as a volcano plot (effect size > 0: higher phosphorylation in EGFRvIII+; significance score > 1.3 indicates significant changes, dotted line). **f** Upstream kinase analysis of EGFRvIII+ vs. EGFRvIII−; top 20 of the differentially regulated kinases (Normalized kinase statistic > 0: higher kinase activity in EGFRvIII+; specificity score > 1.3 (white to red bars): statistically significant changes). **g** Detection of EGFR, pEGFR (Y1173), p38, pp38 (T180/Y182), JNK and pJNK (T183/Y185) by western blot analysis. β-actin served as loading control. Samples were normalized to cell number. For quantification of relative JNK and p38 phosphorylation, relative phosphorylation values of EGFRvIII+ cells were normalized to the relative values of EGFRvIII− cells (mean with S.E.M, *n* = 3; *P*-values are obtained by one-tailed Student’s *t* test, *p < 0.05). **h** Impact of siRNA-mediated p38 knockdown in BS153vIII + cells on MLH1, MSH2 and MSH6 expression after 24, 48, and 72 h. An siRNA against cyclophilin B served as a control. β-actin served as loading control. For quantification of relative MMR protein expression protein levels were normalized to β-actin and to MLH1, MSH2 and MSH6 levels in the control.

As reported earlier, EGFRvIII+ cells display increased activation of ERK1/2-dependent MAPK signaling (Fig. 5a) [20]. We next analyzed, whether increased MAPK signaling is involved in the upregulation of MMR. Indeed, a partial knockdown of ERK1/2 resulted in a downregulation of MLH1 and MSH2 but not MSH6 (Fig. 5b), which was also significantly upregulated in both EGFRvIII+ sub-cell lines (Fig. 4b). To identify additional serine/threonine kinase-dependent downstream pathways involved in EGFRvIII signaling, we performed functional kinomic profiling of BS153vIII−/+ cells using a peptide based in vitro assay (Fig. 5c) [40]. The heat map in Fig. 5d shows increased phosphorylation of several peptides if the arrays were incubated with lysates from EGFRvIII+ cells, which reached significance for 25 peptides (Fig. 5e). The upstream kinase analysis confirmed the increased activation of ERK1/2 and identified additional upregulated MAP-kinases, such as p38, JNK and ERK5 (Fig. 5f). We could not verify upregulated ERK5 activity (data not shown) while phosphorylation of JNK was only increased in BS153vIII+ but not in DKMGvIII+ cells as detected by western blot (Fig. 5g, h). In contrast, we observed increased p38 phosphorylation in both EGFRvIII+ sub-cell lines. A partial siRNA mediated knockdown of p38 resulted in downregulation of all three MMR proteins, MLH1, MSH2, and MSH6 in BS153vIII+ cells (Fig. 5i), demonstrating that besides the ERK1/2-dependent MAPK pathway, also the p38-dependent MAPK pathway is involved in the EGFRvIII mediated upregulation of MMR proteins.

**Fig. 6:**
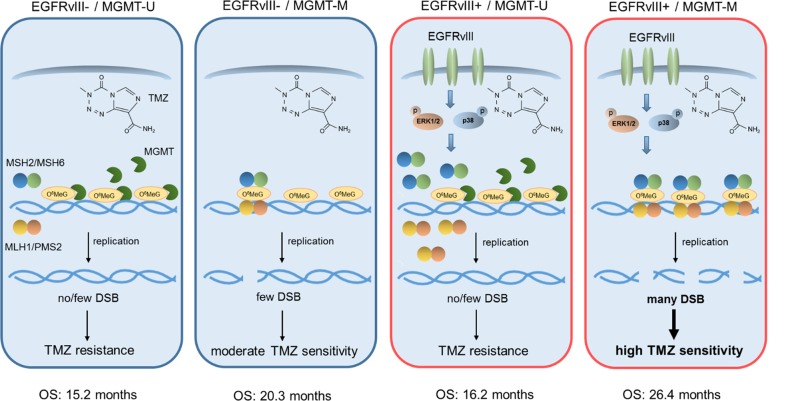
Model. Under conditions where MGMT is expressed (MGMT-U), both EGFRvIII- and EGFRvIII+ GBM cells are resistant to TMZ. This is because MGMT removes the O^6^MeG lesions and therefore prevents replication-dependent induction of DNA DSB. EGFRvIII− cells which do not express MGMT (*MGMT*-M) display moderate TMZ sensitivity, because O^6^MeG lesions are converted through futile MMR cycles into DSB. In MGMT deficient (*MGMT*-M) cells expressing EGFRvIII, MAPK signaling is activated, which leads to upregulation of MMR protein expression. This leads to a more efficient conversion of O^6^MeG lesions into lethal DSB and, consequently, to increased TMZ sensitivity.

In summary, we propose a model, according to which EGFRvIII expression leads to increased TMZ sensitivity in *MGMT* promoter methylated cells through the upregulation of MMR proteins via the ERK1/2 and p38 pathways (Fig. 6). These findings offer an explanation for the observed better prognosis of EGFRvIII+ GBM patients carrying *MGMT* promoter methylation.

## Discussion

EGFRvIII is an oncogene, which is expressed in about 30% of all GBM. Although multiple clinical studies have addressed the prognostic relevance of EGFRvIII, the impact of EGFRvIII expression on treatment response and therefore patient survival is still under controversial debate. We show here that EGFRvIII expression leads to increased TMZ sensitivity in *MGMT* promoter methylated GBM, which strongly indicates an upregulated MMR. If expressed, MGMT removes the O^6-^alkylguanine DNA adduct through covalent transfer of the alkyl group to the conserved active site, thereby restoring the guanine to its normal form. In the absence of MGMT, TMZ-induced O^6^-methylguanine pairs with thymine, leading to a futile MMR repair cycle which ends up in the induction of highly toxic DNA DSB during replication [32, 33, 39]. Therefore, MMR activity determines the sensitivity towards TMZ and other alkylating agents [35, 37, 38], while the level of MMR activity is limited by the expression level of the MMR proteins [41].

In line with that model, we detected increased MMR protein expression in EGFRvIII+ cells and human tumor samples (Fig. 4d, e) demonstrating increased MMR to be the reason for the increased TMZ sensitivity in MGMT-deficient cells and tumors. As expected, the upregulation of MMR protein expression in EGFRvIII+ cells was accompanied by an increase in DSB formation (Fig. 3f). Already small changes in the expression of single MMR proteins had a strong influence on TMZ sensitivity, as demonstrated by partial knockdown experiments (Fig. 4a). This is in line with other studies, which also demonstrated that already moderately altered MMR protein levels can have a profound impact on TMZ sensitivity [37]. This sensitivity of MMR activity towards small changes in the expression of MMR proteins can be explained by the fact that MMR proteins interact pairwise in a 1:1 stoichiometry [37]. Therefore, the least abundant protein determines MMR activity, whereas changes in abundant proteins such as PMS2 (Fig. 4b, c) will hardly be of consequence.

The clinical relevance of this interrelation is underpinned by a study by Felsberg et al. who observed frequent decreases in MMR protein expression in recurrent GBM samples after TMZ treatment relative to their primary tumors [42]. Besides, Yip et al. observed that MSH6 mutations arise in certain GBM under TMZ treatment, leading to TMZ resistance [43]. These data and the data presented here indicate that MMR deficiency and changes in MMR protein expression in GBM are of greater relevance than hitherto assumed.

The increased TMZ sensitivity, which we observed using our isogenic and endogenous EGFRvIII model system, is consistent with previous studies using EGFRvIII+ neurospheres or transfected U87MG cells [15, 44]. Together with our results, showing a profound increase in MMR protein expression in EGFRvIII+ areas of human GBM, all these data strongly indicate elevated MMR to be the cause of the improved survival of *MGMT*-M/EGFRvIII+ patients observed in our retrospective analysis. Further, we could identify the ERK1/2-dependent and the p38-dependent MAPK pathways as regulators of MMR expression in both EGFRvIII+ sub-cell lines (Fig. 5). This is likely due to increased transcription, since MMR genes are regulated by MAPK-dependent transcription factors such as AP1 or SP1 [45, 46]. It is important to state that this upregulation of MAPK was detected in the EGFRvIII+ cells by comparing them to the EGFRvIII− but still EGFR amplified (data not shown) sub-cell lines, demonstrating an EGFRvIII specific activation. Whether other downstream pathways also influence MMR needs to be analyzed in future studies. However, functional kinomics data already hint towards additional MAP kinases, such as JNK and ERK5, although subsequent validation demonstrates that these might not be of relevance in all, but maybe only in individual EGFRvIII expressing GBM (data not shown).

The finding that EGFRvIII+ patients treated with standard of care have a better prognosis compared to EGFRvIII− patients (Fig. 1d, e) agrees with some but also contradicts other studies on the prognostic impact of EGFRvIII (Table S1). Our analysis reveals that it is important to include *MGMT* status to identify the impact of EGFRvIII on therapy outcome: *MGMT*-M/EGFRvIII+ GBM patients showed the most favorable survival after standard of care treatment (Fig. 1d, e). Our data are supported by at least the following studies: (i) Montano et al*.* had also reported a favorable prognosis for EGFRvIII+ and MGMT-M patients [15]; (ii) van den Bent et al*.* observed that approximately one-half of the tumors originally expressing EGFRvIII at initial diagnosis had lost their EGFRvIII expression at tumor recurrence after standard of care treatment [30]; (iii) Weller et al*.* reported an unexpected long survival of EGFRvIII+ patients in the ACTIV trial. While this finding might in principle also be explained by patient selection, they also observed a loss of EGFRvIII expression in the non-vaccinated placebo plus standard of care group [47], which is in line with the observations of van den Bent et al.

In addition to the prognostic value of EGFRvIII, our observation that EGFRvIII expression and MAPK activation result in an increased MMR and therefore TMZ sensitivity, may be of direct clinical relevance because current clinical trials are testing the combination of TMZ with EGFR/EGFRvIII or MAPK targeting approaches or are assessing new EGFRvIII-specific treatment approaches in recurrent GBM (NCT02573324, NCT02364206, NCT03283631). Our data indicate that such combinations may lead to TMZ resistance due to decreased MMR protein expression, or that recurrent GBM might have lost their EGFRvIII expression after initial standard of care treatment. Therefore, anti-EGFRvIII and anti-MAPK strategies should be used very cautiously when combined with TMZ. This finding might also help explain the failure of the EGFR and EGFRvIII targeting trials published so far [48, 49].

In contrast to TMZ sensitivity, radiotherapy outcomes seem to be unaffected by EGFRvIII, because i) we observed no differences in patient survival between the EGFRvIII+ and EGFRvIII− group for *MGMT*-U tumors where TMZ is of less relevance, ii) former clinical trials published before the implementation of TMZ, but including irradiation, showed no survival differences with regard to EGFRvIII expression (table S1) and iii) our previous preclinical data demonstrated no impact of EGFRvIII on cellular radiosensitivity [20].

Altogether, our data strongly suggest that *MGMT* promoter methylation and EGFRvIII are two closely linked biomarkers predicting the outcome under standard of care treatment; together they identify tumors with increased TMZ sensitivity and best prognosis as summarized in Fig. 6. Since approximately 13% of GBM are *MGMT*-M/EGFRvIII+ tumors, based on the three analyzed cohorts, EGFRvIII positivity together with *MGMT* promoter methylation status might be considered as relevant factors, e.g. for patient stratification in future clinical trials. Furthermore, our results highlight that oncogenes, such as EGFRvIII, do not always mediate resistance against radio- or chemotherapy. By upregulating certain DNA repair pathways such as MMR, they can also generate a specific and targetable Achilles´ heel in some tumors that may be further explored in future personalized therapies. With regard to EGFRvIII this might not only be of relevance for adult GBM but also for other tumor entities, such as pediatric brain tumors and ovarian cancers, which also show EGFRvIII expression [50, 51].

## Materials and methods

### Patient survival analysis

Data from 336 GBM patients with IDH1 wild type tumors and known *MGMT* promoter methylation, EGFRvIII, *EGFR* amplification, and *IDH1* status were included in the analysis. *IDH2* mutations were no exclusion criteria since these are rare events in GBM. Patients had all been treated with standard of care, comprised of radiotherapy with concurrent temozolomide, followed by temozolomide alone. Recurrent resection was an exclusion criteria. Survival analysis was performed using the ‘survival’ and ‘survminer’ packages in the R statistical software suite with analysis on overall survival in days. The significance of the separation of Kaplan–Meier survival curves, based on EGFRvIII status, was calculated using a log-rank test. The data originated from three independent cohorts, herein referred to as the University of Pennsylvania (UPenn [28]) cohort (230 cases), the *North Bristol NHS Trust* (Bristol [18]*)* cohort (29 cases) and the TCGA cohort (79 cases, extracted from the Brennan et al. publication [29]).

### University of Pennsylvania patient cohort

GBM Patients undergoing surgical resection for a cranial malignancy were identified through the University of Pennsylvania’s Center for Personalized Diagnostics (CPD). Exclusion criteria were *IDH1* mutation, 1p19q co-deletion, recurrent resection, and missing EGFRvIII or *MGMT* promoter methylation status [28, 52]. For the UPenn cohort IDH1 status was obtained by both IHC staining and confirmatory next generation sequencing (NGS). All UPenn patient data were obtained retrospectively under a protocol approved by the University of Pennsylvania’s Institutional Review Board, with a waiver for patient consent.

### North Bristol NHS Trust

GBM patients were tested for *EGFR* amplification by fluorescence in situ hybridization (FISH) and immunohistochemistry, whereas EGFRvIII expression was detected by reverse-transcription PCR and immunohistochemistry [18]. While known MGMT promoter methylation, EGFRvIII, EGFR amplification were inclusion criteria, exclusion criteria were *IDH1* mutation, 1p19q co-deletion, recurrent resection, and missing EGFRvIII or *MGMT* promoter methylation status. *MGMT* promoter methylation analysis was carried out by bisulphite conversion and pyrosequencing using primers as described by Dunn et al. [53]. *IDH* mutation status was performed by immunohistochemistry for the R132H mutation.

In terms of the Bristol patients, samples were obtained from the Brain Tumour Bank South West (BRASH) at North Bristol NHS Trust UK under approval of Research Ethics Committee for Wales with a waiver for patient consent.

### TCGA Cohort

GBM patient data were extracted from the Brennan et al. publication [29]. For the TCGA cohort IDH status was obtained by NGS or Sanger-based sequencing.

### GBM patient samples

Human tumor material was used in accordance with all local and national ethics guidelines.

### Cell culture

The human GBM cell lines DKMGvIII−/+ and BS153vIII−/+ were generated and characterized as described previously [20]. All cells were identified by a short tandem repeat multiplex assay (Applied Biosystems).

### Xenografts

Nine-week old female BALB/c nude mice (BALB/cAnNCrl-Foxn1<nu>) were obtained from Charles River UK. 2 × 10^5^ BS153vIII−/+ cells in 2 µl DMEM were injected into the right frontal lobe of each animal using a stereotactic frame. Mice were randomly assigned to experimental groups (10 mice/group) for treatment, which commenced two weeks after the surgery. TMZ was administered by intraperitoneal (IP) injection at 5 mg/kg/day in 10% DMSO for five consecutive days. Control animals received 10% DMSO in PBS, IP, following the same schedule. Animal welfare was monitored and documented daily and mice were euthanized when they exhibited neurological symptoms and/or substantial weight loss (maximum tolerated loss was 20% of body mass). Animals were culled by perfusion/fixation (4% paraformaldehyde; PFA) under terminal anaesthesia. All work was performed in accordance with the United Kingdom Animals (Scientific Procedures) Act (1986), under the authority of project licence 70/7340. Kaplan–Meier curves were generated in GraphPad Prism and statistical analysis of survival was generated automatically by the software using log-rank test.

### Reagents

Temozolomide (stock concentration: 100 mM, Sigma-Aldrich) was dissolved in DMSO (Sigma-Aldrich) and stored at −20 °C. Staurosporine (stock concentration: 1 mM, Sigma-Aldrich), stored at −20 °C.

### PCR *MGMT* promoter methylation

*MGMT* promoter methylation was determined by methylation-specific PCR (MSP). The isolation of nucleic acids, bisulphite modification of DNA and methylation-specific PCR (MSP) were performed following standard laboratory protocols and using commercially available kits performed according to the manufacturer’s protocols.

### Colony forming assay

To analyze the ability of GBM cells for self-renewal (clonogenicity) 200–350 cells were seeded 24 h prior to treatment with TMZ. After 24 h of treatment the medium was replaced by AmnioMax C-100 containing 10% FCS and C-100 supplement (Life Technologies) to allow proper colony formation. All cells were cultured at 37 °C, 5% CO_2_ and 100% humidification for colony growth. The cells were grown until the colonies of all treatment arms had reached equal colony size (approximately 14–28 d). The cells were fixed, stained and colonies of more than 50 cells were counted manually. The surviving fraction of treated cells was normalized to the plating efficiency of non-treated cells.

### siRNA mediated knockdown

Knockdown of EGFRvIII, MLH1, MSH6, ERK1/2, and p38 was performed using HiPerFect (Qiagen, #301705) according to the manufacturer´s instructions. The following siRNAs were used: EGFRvIII from Eurofins Scientific; [5´-CUGGAGGAAAAGAAAGGUAAU-3´]; MLH1 and MSH6 (Ambion, #AM51331; #AM1678); ERK1/2 siRNA (Cell Signaling, #6560), p38 siRNA (Cell Signaling, #6243). On-Target plus Cyclophilin B control pool as control siRNA (Dharmacon, #SO-2436533G). The medium was changed 5 h after transfection and 24 h later the colony forming assay was performed as described below.

### Western blot

Proteins from whole-cell extracts were detected by Western blot according to standard protocols. The *Odyssey® CLx Infrared Imaging System* (LI-COR Biosciences) was used for signal detection and quantification. Primary antibodies: EGFR (1:1000, rabbit, Cell Signaling, #2232); pEGFR (1:1000, rabbit, Cell Signaling, #4407); β-Actin (1:20,000, mouse, Sigma-Aldrich, #A-2228); MLH1 (1:1000, Cell Signaling, #3515); MSH2 (1:1000, Cell Signaling, #2850); MSH6 (1:1000, Cell Signaling, #4407); MSH3 (1:1000, BD Transduction Laboratories, #611390); PMS2 (1:1000, Rockland, #29379); PCNA (1:1000, Santa Cruz Biotechnology, sc-56); ERK1/2 (1:1000. Cell Signaling, #9107); pERK1/2 (1:1000. Cell Signaling, #4370); p38 (1:1000, Cell Signaling, #8690); pp38 (1:1000, Cell Signaling, #4511); JNK (1:1000. Cell Signaling, #9252), pJNK (1:1000. BD Transduction Laboratories, #612540). All primary antibodies were diluted in 5% bovine serum albumine (BSA) in PBS supplemented with 0.2% Tween. Secondary anti-mouse and anti-rabbit antibodies were purchased from LI-COR Biosciences.

### Flow cytometry

Flow cytometry was performed as described previously using a FACSCanto and FACSDiva software (BD Biosciences) [20, 54]. For EGFRvIII detection and quantification anti-EGFRvIII antibody L8A4 (1:1000, mouse, Absolute antibody, #Ab00184-1.4) and Alexa fluor® 647 labeled secondary antibody (1:1000, Life Technologies, #A-21235) were usd. Cell-surface exposure of phosphatidylserine was assessed using an Annexin-V-FITC conjugate following the manufacturer’s instructions (Thermo Scientific, #A13199). For cell cycle analysis via propidium iodid staining the fraction of G1, S, and G2 phase cells was calculated using ModFit LT™ software (Verity Software House, Inc.).

### Immunofluorescence staining

EGFRvIII specific immunofluorescence was performed as reported earlier [20]. For DNA repair analysis cells were fixed, blocked and stained with the anti-53BP1 (1:1000, Novus, Biologicals, #NB-100-3004) and γH2AX (1:500, MerckMilipore, #05-636) antibody and the respective secondary antibodies (ALEXA fluor® 488; ALEXA fluor® 594, 1:1000, Life Technologies, #A-11001; A-11005) at RT for 60 min; DNA was then stained with 4′,6-diamidino-2-phenylindole (DAPI; QBiogene). A confocal fluorescence microscope (Zeiss Axio Observer Z1; 630x magnification) was used for analysis. At least 100 intact nuclei were randomly selected and γH2AX/53BP1 double positive foci were counted. Z-stacks of semi-confocal images were obtained using the Zeiss Apotome, Zeiss AxioCam MRm and Zeiss AxioVision Software and 53BP1 foci were counted using ImageJ.

### Immunohistochemistry

Five-micrometer sections of paraffin-embedded GBM specimens were dewaxed using standard histologic procedures. Heat-induced antigen retrieval for the detection of MSH2 (1:1000; rabbit, Abcam #ab72070) and MSH6 (1:250; mouse, Dako, #3646) was carried out by boiling slides in sodium citrate buffer, pH 6.8, for 1 hour. Primary antibodies were incubated overnight at 4 °C. Specimens were then incubated with secondary antibody (Immpress reagent kit anti-rabbit IgG, Vector Laboratories, #MP7401 or Immpress reagent kit anti-mouse IgG, Vector Laboratories, MP7402) for 1 h. Bound secondary antibody was detected by ImmPact DAB substrate (Vector Laboratories, #sk-405). EGFRvIII staining was performed on a Ventana System using standard protocols (1:250, mouse, Absolute antibody, #Ab00184-1.4). Nuclei were counterstained with hematoxylin. After staining the specimes werde dehydrated covered using standard histologic procedures.

### Kinase activity profiling

Kinase activity profiling has been described previously [40]. Here we used a PamStation®12 (located at the UCCH Kinomics Core Facility, UKE, Hamburg, Germany) according to the manufacturer’s instructions (PamGene International, ´s-Hertogenbosch, The Netherlands). In brief, whole-cell lysates were made using M-PER Mammalian Extraction Buffer containing Halt Phosphatase Inhibitor and EDTA-free Halt Protease Inhibitor Cocktail (Pierce, Waltham, Massachusetts, USA). For profiling serine/threonine kinases, STK-PamChip® arrays were incubated with 1 μg of protein and 400 μM ATP per array. Each array contains 140 individual phospho-sites that are peptide sequences derived from substrates for serine/threonine kinases. Sequence-specific peptide serine/threonine phosphorylation was detected in two steps, first with anti-phospho-Ser/Thr antibodies during the reaction followed by detection with secondary antibody (polyclonal swine anti-rabbit Immunoglobulin/FITC, PamGene International). Signals were recorded using a CCD camera and the Evolve software (PamGene International, ´s-Hertogenbosch, The Netherlands). After quality control, the final signal intensities were log2-transformed and were used for further data analysis using the BioNavigator software version 5.1 (PamGene International, ‘s-Hertogenbosch, The Netherlands).

### Statistical analysis (pre-clinical data)

Except for tumor cell injection and unless otherwise indicated, experiments were repeated at least three times. The data are presented as mean values (±SEM) except for the patient data. Prism software (GraphPad Prism 5, GraphPad Software, Inc.) was used for analyzing and graphing the data. *P*-values were calculated using Student´s *t*-tests (*p < 0.05; **p < 0.01; ***p < 0.001).

## Supplementary information

supplementary table S1

supplementary material

supplementary table S2
