# Supplementary material for: EGFRvIII upregulates DNA mismatch repair resulting in increased temozolomide sensitivity of MGMT promoter methylated glioblastoma
Source: Oncogene. 2020 Feb 17;39(15):3041–55. doi: 10.1038/s41388-020-1208-5 (PMC7142016; doi:10.1038/s41388-020-1208-5)
Supplement: Supplementary file 1 — supplementary table S1 [file 41388_2020_1208_MOESM1_ESM.docx]

**Table S1:** Summary of studies analyzing the clinical impact of EGFRvIII

| **Author** | **Year** | **Patients (N)** | **treatment** | **Outcome** |
| --- | --- | --- | --- | --- |
| Feldkamp et al.^(^[^1^](#_ENREF_1)^)^ | 1999 | 12 | surgery | worse |
| Shinojima et al. ^(^[^2^](#_ENREF_2)^)^ | 2003 | 87 | surgery (GTR, PR, biopsy), ACNU, PCB | worse |
| Aldape et al.^(^[^3^](#_ENREF_3)^)^ | 2004 | 105 | surgery, RT, CT (N/S) | not prognostic |
| Heimberger et al.^(^[^4^](#_ENREF_4)^)^ | 2005 | 196 | RT | not prognostic (worse prognosis for patients surviving over 1 year) |
| Liu et al. ^(^[^5^](#_ENREF_5)^)^ | 2005 | 160 | surgery, 50% received RT | not prognostic |
| Heimberger et al.^(^[^6^](#_ENREF_6)^)^ | 2005 | 54 | surgery (GTR, PR, biopsy), RT, chemo (N/S) | not prognostic |
| Mellinghoff et al.^(^[^7^](#_ENREF_7)^)^ | 2005 | 49 | erlotinib, gefitinib | Better prognosis in the erlotinib arm, if patients were EGFRvIII positive and PTEN deleted |
| Pelloski et al.^(^[^8^](#_ENREF_8)^)^ | 2007 | 509 | surgery, RT | worse |
| Viana-Pereira et al.^(^[^9^](#_ENREF_9)^)^ | 2008 | 27 | N/S | not prognostic |
| Brown et al.^(^[^10^](#_ENREF_10)^)^ | 2008 | 81 | surgery (GTR, SBT, biopsy) RT, TMZ, erlotinib | not prognostic |
| Van den Bent et al.^(^[^11^](#_ENREF_11)^)^ | 2009 | 49 | erlotinib, BCNU, TMZ | not prognostic, worse in erlotinib arm |

| Montano et al.^(^[^12^](#_ENREF_12)^)^ | 2011 | 73 | surgery (GTR, PR), RT, TMZ | better |
| --- | --- | --- | --- | --- |
| Bienkowski et al.^(^[^13^](#_ENREF_13)^)^ | 2013 | 58 | surgery, RT, chemo (N/S) | better |
| Weller et al.^(^[^14^](#_ENREF_14)^)^ | 2014 | 179 | surgery, RT, TMZ | not prognostic |
| Faulkner et al. ([15](#_ENREF_15)) | 2014 | 50 | surgery, RT, TMZ | not prognostic |
| Cominelli et al. ([16](#_ENREF_16)) | 2015 | 44 | surgery, RT, TMZ (SS+MS) | not prognostic |
| Felsberg et al. ([17](#_ENREF_17)) | 2017 | 106 | surgery, RT, TMZ | not prognostic |

GTR, gross total resection; PR, partial resection; ACNU, 3-[(4-amino-2-methyl-5-pyrimidinyl)methyl]-1-(2-chloroethyl)-1-nitrosourea hydrochloride; PCB, procarbazine, RT, radiotherapy; CT, chemotherapy; N/S, not specified; SBT, sub total resection, BCNU, bis-chloroethylnitrosourea; SBT, sub total resection; SS, standard schedule, MS, metronomic schedule

**Supplementary References**

1. Feldkamp MM, Lala P, Lau N, Roncari L, Guha A. Expression of activated epidermal growth factor receptors, Ras-guanosine triphosphate, and mitogen-activated protein kinase in human glioblastoma multiforme specimens. Neurosurgery. 1999 Dec;45(6):1442-53. PubMed PMID: 10598712.

2. Shinojima N, Tada K, Shiraishi S, Kamiryo T, Kochi M, Nakamura H, et al. Prognostic value of epidermal growth factor receptor in patients with glioblastoma multiforme. Cancer research. 2003 Oct 15;63(20):6962-70. PubMed PMID: 14583498.

3. Aldape KD, Ballman K, Furth A, Buckner JC, Giannini C, Burger PC, et al. Immunohistochemical detection of EGFRvIII in high malignancy grade astrocytomas and evaluation of prognostic significance. Journal of neuropathology and experimental neurology. 2004 Jul;63(7):700-7. PubMed PMID: 15290895.

4. Heimberger AB, Hlatky R, Suki D, Yang D, Weinberg J, Gilbert M, et al. Prognostic effect of epidermal growth factor receptor and EGFRvIII in glioblastoma multiforme patients. Clinical cancer research : an official journal of the American Association for Cancer Research. 2005 Feb 15;11(4):1462-6. PubMed PMID: 15746047.

5. Liu L, Backlund LM, Nilsson BR, Grander D, Ichimura K, Goike HM, et al. Clinical significance of EGFR amplification and the aberrant EGFRvIII transcript in conventionally treated astrocytic gliomas. Journal of molecular medicine. 2005 Nov;83(11):917-26. PubMed PMID: 16133418. Pubmed Central PMCID: 2815848.

6. Heimberger AB, Suki D, Yang D, Shi W, Aldape K. The natural history of EGFR and EGFRvIII in glioblastoma patients. Journal of translational medicine. 2005 Oct 19;3:38. PubMed PMID: 16236164. Pubmed Central PMCID: 1298339.

7. Mellinghoff IK, Wang MY, Vivanco I, Haas-Kogan DA, Zhu S, Dia EQ, et al. Molecular determinants of the response of glioblastomas to EGFR kinase inhibitors. The New England journal of medicine. 2005 Nov 10;353(19):2012-24. PubMed PMID: 16282176.

8. Pelloski CE, Ballman KV, Furth AF, Zhang L, Lin E, Sulman EP, et al. Epidermal growth factor receptor variant III status defines clinically distinct subtypes of glioblastoma. Journal of clinical oncology : official journal of the American Society of Clinical Oncology. 2007 Jun 1;25(16):2288-94. PubMed PMID: 17538175.

9. Viana-Pereira M, Lopes JM, Little S, Milanezi F, Basto D, Pardal F, et al. Analysis of EGFR overexpression, EGFR gene amplification and the EGFRvIII mutation in Portuguese high-grade gliomas. Anticancer research. 2008 Mar-Apr;28(2A):913-20. PubMed PMID: 18507036.

10. Brown PD, Krishnan S, Sarkaria JN, Wu W, Jaeckle KA, Uhm JH, et al. Phase I/II trial of erlotinib and temozolomide with radiation therapy in the treatment of newly diagnosed glioblastoma multiforme: North Central Cancer Treatment Group Study N0177. Journal of clinical oncology : official journal of the American Society of Clinical Oncology. 2008 Dec 1;26(34):5603-9. PubMed PMID: 18955445. Pubmed Central PMCID: 2651097.

11. van den Bent MJ, Brandes AA, Rampling R, Kouwenhoven MC, Kros JM, Carpentier AF, et al. Randomized phase II trial of erlotinib versus temozolomide or carmustine in recurrent glioblastoma: EORTC brain tumor group study 26034. Journal of clinical oncology : official journal of the American Society of Clinical Oncology. 2009 Mar 10;27(8):1268-74. PubMed PMID: 19204207. Pubmed Central PMCID: 2667826.

12. Montano N, Cenci T, Martini M, D'Alessandris QG, Pelacchi F, Ricci-Vitiani L, et al. Expression of EGFRvIII in glioblastoma: prognostic significance revisited. Neoplasia. 2011 Dec;13(12):1113-21. PubMed PMID: 22241957. Pubmed Central PMCID: 3257186.

13. Bienkowski M, Piaskowski S, Stoczynska-Fidelus E, Szybka M, Banaszczyk M, Witusik-Perkowska M, et al. Screening for EGFR amplifications with a novel method and their significance for the outcome of glioblastoma patients. PloS one. 2013;8(6):e65444. PubMed PMID: 23762372. Pubmed Central PMCID: 3675194.

14. Weller M, Kaulich K, Hentschel B, Felsberg J, Gramatzki D, Pietsch T, et al. Assessment and prognostic significance of the epidermal growth factor receptor vIII mutation in glioblastoma patients treated with concurrent and adjuvant temozolomide radiochemotherapy. International journal of cancer Journal international du cancer. 2014 May 15;134(10):2437-47. PubMed PMID: 24614983.

15. Faulkner C, Palmer A, Williams H, Wragg C, Haynes HR, White P, et al. EGFR and EGFRvIII analysis in glioblastoma as therapeutic biomarkers. British journal of neurosurgery. 2014 Aug 20:1-7. PubMed PMID: 25141189.

16. Cominelli M, Grisanti S, Mazzoleni S, Branca C, Buttolo L, Furlan D, et al. EGFR amplified and overexpressing glioblastomas and association with better response to adjuvant metronomic temozolomide. Journal of the National Cancer Institute. 2015 May;107(5). PubMed PMID: 25739547.

17. Felsberg J, Hentschel B, Kaulich K, Gramatzki D, Zacher A, Malzkorn B, et al. Epidermal Growth Factor Receptor Variant III (EGFRvIII) Positivity in EGFR-Amplified Glioblastomas: Prognostic Role and Comparison between Primary and Recurrent Tumors. Clinical cancer research : an official journal of the American Association for Cancer Research. 2017 Nov 15;23(22):6846-55. PubMed PMID: 28855349.
