# Supplementary material for: EGFRvIII upregulates DNA mismatch repair resulting in increased temozolomide sensitivity of MGMT promoter methylated glioblastoma
Source: Oncogene. 2020 Feb 17;39(15):3041–55. doi: 10.1038/s41388-020-1208-5 (PMC7142016; doi:10.1038/s41388-020-1208-5)
Supplement: Supplementary file 2 — supplementary material [file 41388_2020_1208_MOESM2_ESM.docx]

**Fig. S1**

Fig. S1: Overall survival stratified for GBM patients above and below the median age irrespective of EGFRvIII and MGMT status. Kaplan–Meier estimates of GBM patients treated with standard of care in the population below (B) and the population above (A) the median age.

**Fig. S2**

Fig. S2: Overall survival according to EGFRvIII and MGMT status. Kaplan–Meier estimates of GBM patients treated with standard of care in the *MGMT*-U population and the *MGMT*-M population stratified for EGFRvIII status.

**Fig. S3**

Fig. S3: Overall survival according to EGFRvIII and MGMT status only in *egfr* amplified GBM patients. Kaplan–Meier estimates of GBM patients treated with standard of care in the *MGMT*-U population and the *MGMT*-M population stratified for EGFRvIII status.

Fig. S4

***EGFRampl.***

Fig. S4: Overall survival according to EGFRvIII and *EGFR*ampl. status. Kaplan–Meier estimates of GBM patients treated with standard of care in the *EGFR*ampl. population.

**Fig. S5**

**a**

**b**

**Fig. S5: MGMT expression in DKMGvIII-/+ and BS153vIII-/+ is not changed after TMZ treatment.**a) DKMGvII- /+ or b) BS153vIII-/+ were treated with DMSO as a control (-) or TMZ (+) for 24, 48 or 72 h. Detection of MGMT expression by Western blot analysis. MGMT expressing Jurkat cells (JC) served as a positive and β-Actin as loading control.

**Fig. S6**

**Fig. S6: EGFR and EGFRvIII expression in BS153vIII-/+ cells at the time point of intracranial injection.** Detection of EGFRvIII expression in BS153vIII-/+ sub lines as detected by flow cytometry (dot plots). Almost no EGFRvIII expression in BS153vIII- was detectable in BS153vIII- cells, whereas over 90% of the BS153vIII+ cells expressed EGFRvIII. EGFRvIII expression (APC-A) was detected using an EGFRvIII-specific antibody (L8A4). A secondary antibody control was used to asses unspecific staining.

**Fig. S7**

**Fig. S7: Overall survival of BS153vIII- and BS153vIII+ tumor xenografts.** Two weeks after intracranial injection of 2.5 x 10^5^ BS153vIII- respectively BS153vIII+ cells, mice were treated with 10% DMSO/PBS as a control for 5 d. A tendency to shorter survival in the BS153vIII+ group was observed, but this difference did not reach statistical significance. (Kaplan-Meier analysis, log rank test, p = 0.0647).

**Fig. S8**

**Fig. S8: Cell cycle distribution after DMSO and TMZ treatment analyzed by PI staining.** G2: grey; S: light grey; G1: white.
