# Supplementary material for: EGFRvIII upregulates DNA mismatch repair resulting in increased temozolomide sensitivity of MGMT promoter methylated glioblastoma
Source: Oncogene. 2020 Feb 17;39(15):3041–55. doi: 10.1038/s41388-020-1208-5 (PMC7142016; doi:10.1038/s41388-020-1208-5)
Supplement: Supplementary file 3 — supplementary table S2 [file 41388_2020_1208_MOESM3_ESM.docx]

**Tab.S2:** *MGMT* promotor methylation and EGFR amplification status of EGFRvIII negative and EGFRvIII positive GBM patients

| UPenn cohort^(24)^ | all patients (*n*=230)  *n* (%) | EGFRvIII negative *(n*=168)  *n* (%) | EGFRvIII positive (*n*=62)  *n* (%) |
| --- | --- | --- | --- |
| *MGMT* promotor |  |  |  |
| unmethylated | 135 (59) | 102 (61) | 33 (53) |
| methylated | 95 (41) | 66 (39) | 29 (47) |
| *EGFR* |  |  |  |
| unamplified | 133 (58) | 125 (74) | 8 (13) |
| amplified | 97 (42) | 43 (26) | 54 (87) |
|  |  |  |  |
| Bristol cohort^(22)^ | **all patients (*n*=27)**  ***n* (%)** | **EGFRvIII negative *(n*=16)**  ***n* (%)** | **EGFRvIII positive (*n*=11)**  ***n* (%)** |
| *MGMT* promotor |  |  |  |
| unmethylated | 13 (48) | 8 (50) | 5 (45) |
| methylated | 14 (52) | 8 (50) | 6 (55) |
| *EGFR* |  |  |  |
| unamplified | 12 (44) | 10 (63) | 2 (18) |
| amplified | 15 (56) | 6 (37) | 9 (82) |
|  |  |  |  |
| TCGA cohort^(25)^ | **all patients (*n*=79)**  ***n* (%)** | **EGFRvIII negative *(n*=60)**  ***n* (%)** | **EGFRvIII positive (*n*=19)**  ***n* (%)** |
| *MGMT* promotor |  |  |  |
| unmethylated | 41 (52) | 31(52) | 10 (53) |
| methylated | 38 (48) | 29 (48) | 9 (47) |
| *EGFR* |  |  |  |
| unamplified | 5 (6) | 4 (7) | 1 (5) |
| amplified | 74(94) | 56 (93) | 18 (95) |
|  |  |  |  |
| combined cohort | **all patients (*n*=336)**  ***n* (%)** | **EGFRvIII negative *(n*=244)**  ***n* (%)** | **EGFRvIII positive (*n*=92)**  ***n* (%)** |
| *MGMT* promotor |  |  |  |
| unmethylated | 189 (56) | 140 (57) | 48 (52) |
| methylated | 147 (44) | 104 (43) | 44 (48) |
| *EGFR* |  |  |  |
| unamplified | 150 (45) | 139 (57) | 11 (12) |
| amplified | 186 (55) | 105 (43) | 81 (88) |
